# Supplementary material for: Resolving the full spectrum of human genome variation using Linked-Reads
Source: Genome Res. 2019 Apr;29(4):635–45. doi: 10.1101/gr.234443.118 (PMC6442396; doi:10.1101/gr.234443.118)
Supplement: Supplemental Material [file supp_gr.234443.118_Supplemental_longranger-2.2.zip › longranger-2.2.tar.bz2!/lr2.2-public/lib/stan/cmdstan/stan/doc/api/html/globals_func.html]

Stan: File Members


|  |  |
| --- | --- |
|  | Stan  2.5.0  probability, sampling & optimization |


- Main Page
- Related Pages
- Namespaces
- Classes
- Files

- File List
- File Members

- All
- Functions
- Variables
- Macros

- b
- c
- d
- i
- m
- n
- p
- s


All Classes Namespaces Files Functions Variables Typedefs Enumerations Enumerator Friends Macros Pages

### - b -

- base\_param\_name()
  : print.hpp
- BOOST\_FUSION\_ADAPT\_STRUCT()
  : bare\_type\_grammar\_def.hpp
  , functions\_grammar\_def.hpp
  , program\_grammar\_def.hpp
  , statement\_grammar\_def.hpp
  , term\_grammar\_def.hpp
  , var\_decls\_grammar\_def.hpp

### - c -

- calculate\_column\_width()
  : print.hpp
- calculate\_column\_widths()
  : print.hpp
- compute\_precision()
  : print.hpp
- compute\_width()
  : print.hpp
- compute\_width\_and\_precision()
  : print.hpp

### - d -

- delete\_file()
  : stanc\_helper.hpp
- dimensions()
  : print.hpp

### - i -

- is\_matrix()
  : print.hpp

### - m -

- matrix\_index()
  : print.hpp

### - n -

- next\_index()
  : print.hpp

### - p -

- print\_stanc\_help()
  : stanc\_helper.hpp
- print\_usage()
  : print.hpp
- print\_version()
  : stanc\_helper.hpp

### - s -

- stanc\_helper()
  : stanc\_helper.hpp


---

[ Stan Home Page ]

*© 2011–2014,
Stan Development Team.*
